# Geospatial suitability of cacao in Amazonas (Peru): An ensemble predictive modeling approach (BIOMOD2) integrating multisource data, territorial management, and plant health

**DOI:** 10.1371/journal.pone.0360376

**Published:** 2026-10-09

**Authors:** Jhon A. Zabaleta-Santisteban, Angel J. Medina-Medina, Katerin M. Tuesta-Trauco, Abner S. Rivera-Fernandez, Teodoro B. Silva-Melendez, Marlen A. Grandez-Alberca, Julio Puscan-Rojas, Rolando Salas López, Manuel Oliva-Cruz, Alexander Cotrina-Sanchez, Nilton B. Rojas-Briceño, Jhonsy O. Silva-López, Darwin Gómez-Fernández, Santos Triunfo Leiva-Espinoza, Angel F. Huaman-Pilco, Elgar Barboza

**Affiliations:** 1 Instituto de Investigación para el Desarrollo Sustentable de Ceja de Selva (INDES-CES), Universidad Nacional Toribio Rodríguez de Mendoza de Amazonas, Chachapoyas, Peru; 2 Grupo de Investigación en Ciencia de la Información Geoespacial (CIGEO), Instituto de Investigación para el Desarrollo del Perú, Universidad Nacional de Moquegua, Pacocha, Peru; 3 Área de cartografía y teledetección, Laboratorio de Agrostología, Instituto de Investigación en Ganadería y Biotecnología, Facultad de Ingeniería Zootecnista, Agronegocios y Biotecnología, Universidad Nacional Toribio Rodríguez de Mendoza de Amazonas, Chachapoyas, Peru; 4 Centro Experimental Yanayacu, Dirección de Servicios Estratégicos Agrarios (DSEA), Instituto Nacional de Innovación Agraria (INIA), Cajamarca, Peru; 5 Facultad de Ingeniería Ambiental, Biosistemas y de la Energía (FIABE), Universidad Nacional Toribio Rodríguez de Mendoza de Amazonas, Chachapoyas, Peru; University of the West Indies, TRINIDAD AND TOBAGO

## Abstract

Identifying climatically suitable areas for cacao cultivation (Theobroma cacao L.) is essential to guide sustainable intensification, territorial planning, and compliance with emerging environmental standards in tropical regions. This study assessed the current climatic suitability of cacao in the Amazonas department (northwestern Peru) using an ensemble modeling approach implemented in biomod2, integrating bioclimatic, topographic, and edaphic predictors with spatial biophysical exclusion criteria, legal conservation constraints, regulatory eligibility associated with the European Union Deforestation-Free Products Regulation (EUDR), and local phytosanitary evidence. A total of 403 quality-controlled presence records were used to calibrate multiple machine-learning algorithms under cross-validation, retaining only those with high predictive performance (AUC > 0.9; TSS > 0.7). Decision tree–based models, particularly Random Forest and XGBoost, showed the best individual performance, while the consensus model exhibited greater spatial stability and overall accuracy. Results indicate that approximately 14.8% of the regional territory presents high climatic suitability under current conditions, concentrated in inter-Andean valleys and low- to mid-elevation zones, with thermal seasonality identified as the main environmental predictor. However, the incorporation of territorial and regulatory constraints substantially reduced the effectively available area, especially under the EUDR scenario, demonstrating that cacao expansion potential is strongly conditioned by conservation and regulatory frameworks. High-priority degraded areas emerged as focal opportunities for productive restoration through agroforestry systems. Phytosanitary validation (2021–2024) revealed high spatial concordance between areas of greatest climatic suitability and the prevalence and incidence of major cacao pathogens and pests, particularly *Moniliophthora roreri* and *M. perniciosa*, confirming that optimal climatic niches for cultivation also favor biotic pressure. Overall, this study provides an integrative framework combining climatic modeling, territorial management, and phytosanitary risk, delivering robust scientific inputs to inform public policy, land-use planning, and sustainable cacao production strategies in the Amazon.

## Introduction

Cacao (*Theobroma cacao L.*) is a strategic perennial crop in the tropics due to its economic importance and its contribution to rural livelihoods [1]. Globally, its value chain supports millions of people, with approximately 5 million producing families, predominantly smallholders, and an estimated cultivated area of about 11 million hectares across the humid tropics [2–4]. Nevertheless, persistent constraints remain, including low yields, technological gaps, market vulnerability, and high biotic pressure from pests and diseases [5].

Global supply remains strongly territorially concentrated, increasing the sensitivity of the value chain to environmental and economic disturbances [6]. In 2022, global production reached approximately 5,047,000 t (metric tons), with Africa contributing about 73.6% and the Americas around 20% [6]. This spatial dependence, together with heightened socio-environmental scrutiny, reinforces the need for verifiable geospatial information to support sustainable production management [7–10].

In Peru, cacao is produced predominantly under smallholder Amazonian farming systems and has consolidated as a relevant export commodity [11,12]. The country is also recognized for fine-flavor or aroma cacao, with approximately 75% of its exports reported within this premium segment [6]. Between 2019 and 2023, cacao ranked ninth in terms of contribution to the gross value of agricultural production, with annual volumes estimated at 141.8–166.7 thousand tons (3.3% of agricultural GDP) [13]. In 2023, the average export price reached USD 3.17/kg (+34% year-on-year), consistent with growing demand for differentiated products [14].

Peruvian cacao expansion has occurred in landscapes where the agricultural frontier has historically interacted with tropical forests, generating tensions between production, deforestation, and biodiversity conservation [15,16]. In this context, cacao is crucial for rural livelihoods, including Indigenous territories and peasant communities, and its performance depends primarily on smallholders [17]. Consequently, delineating areas for sustainable intensification and conservation-compatible expansion constitutes a strategic priority [16].

At the national level, the use of GIS, remote sensing, and agricultural suitability approaches has enabled the identification of potentially favorable areas for cacao cultivation, highlighting the Amazonian region as a core area for sustainable cacao production [18,19]. In particular, Amazonas exhibits high ecological and topographic heterogeneity—from high Andean gradients to upper and lower rainforest—configuring mosaics of microclimates and edaphic conditions relevant to diverse production systems [19]. In 2024, Amazonas recorded 4,124 tons, ranking as the seventh cacao-producing department nationwide and consolidating its status as an emerging region [20].

Phytosanitary pressure constitutes a critical risk component, with frosty pod rot (*Moniliophthora roreri*) and witches’ broom (*M. perniciosa*) standing out as widely documented limiting factors in the region [21,22]. Available evidence indicates that their potential distribution is modulated by environmental conditions, justifying the integration of the sanitary component into spatially based territorial assessments [23,24]. Recent reviews on management and biocontrol in Latin America further emphasize the need for preventive approaches supported by geospatial evidence [25].

Species distribution models (SDMs) allow estimation of present-day environmental suitability for cacao by integrating occurrence records with bioclimatic, edaphic, and topographic predictors, generating probabilistic suitability surfaces for GIS analyses [26,27]. Ensemble modeling approaches have gained prominence for improving predictive robustness and reducing uncertainty associated with individual models [26,28]. Within this framework, biomod2 facilitates the calibration and validation of multiple algorithms under a reproducible workflow, suitable for spatial applications [29–31].

Despite these advances, regional analyses that simultaneously integrate ensemble-based SDM suitability, biophysical and conservation constraints, territorial accessibility, and local evidence of phytosanitary pressure remain scarce, particularly in emerging regions such as Amazonas [18,25]. These gaps become more critical under Regulation (EU) 2023/1115, which requires traceability and proof of absence of deforestation and forest degradation after 31 December 2020 [32,33]. Accordingly, consistent geospatial information is required to guide expansion and intensification without compromising forests and biodiversity, while ensuring verifiability for supply chains [16,32].

In this context, the objective of this study is to model the current environmental suitability of cacao in Amazonas using an ensemble approach implemented in biomod2, integrating bioclimatic, topographic, and edaphic predictors with layers of accessibility, conservation status, land degradation, and local evidence of phytosanitary pressure. The results are expected to provide spatial inputs for land-use planning, prioritization of areas for sustainable intensification, and preventive management of sanitary risk under present conditions, in alignment with sustainability and traceability standards.

## Materials and methods

### Study area

The department of Amazonas, located in northwestern Peru (Fig 1), lies within a transition zone between the Andean cordillera and the Amazonian lowlands. This ecotonal position and its physiographic configuration explain the high ecological and geomorphological complexity of the territory [34,35]. Amazonas covers approximately 42,050 km^2^ and is administratively organized into seven provinces—Bagua, Bongará, Chachapoyas, Condorcanqui, Luya, Rodríguez de Mendoza, and Utcubamba—according to the national political-administrative division [36]. The altitudinal range extends approximately from 152 to 5,540 m a.s.l., generating an environmental gradient from lowland rainforest conditions to high Andean mountain environments [37,38].

**Fig 1:**
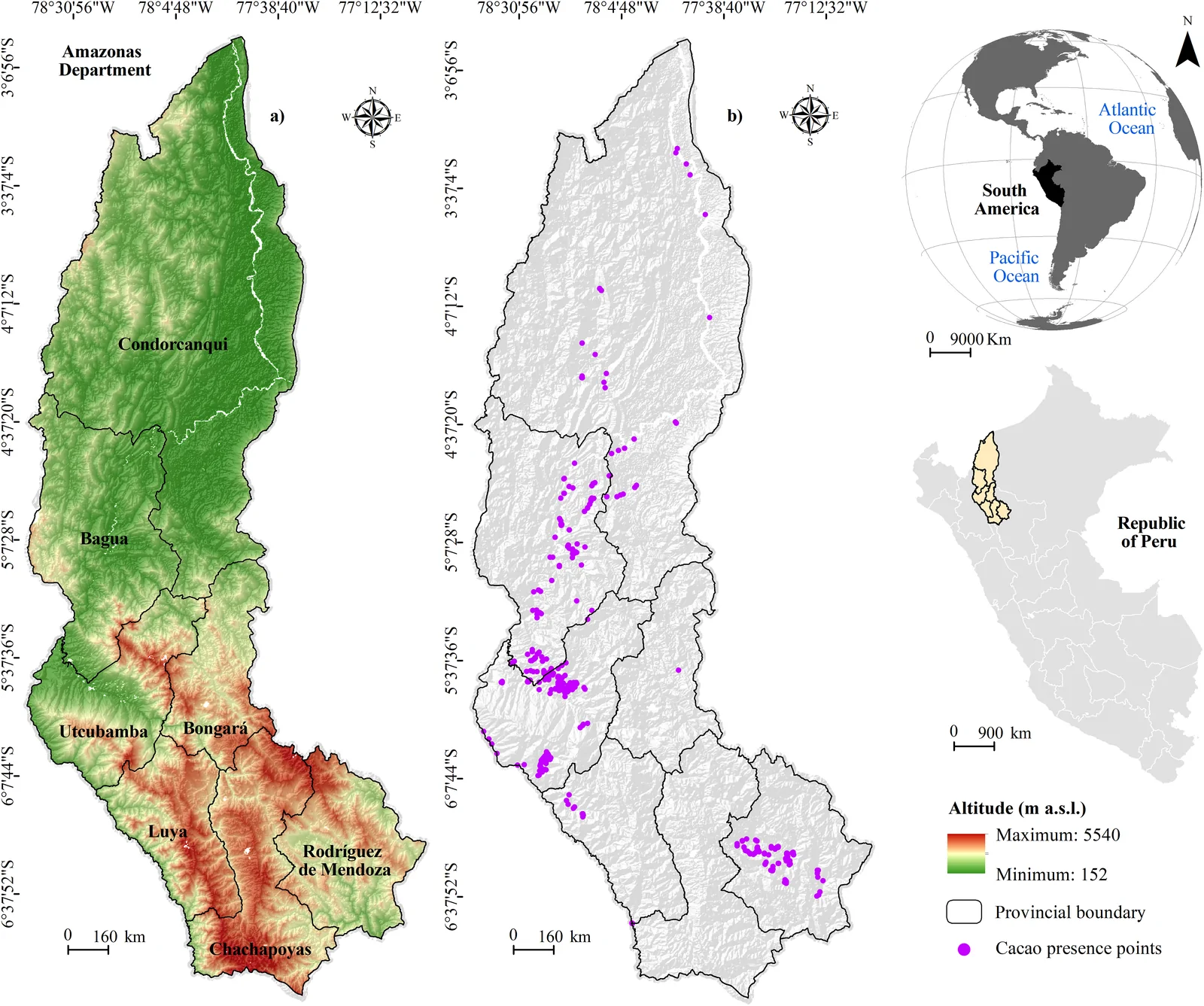
Study area and cacao occurrence records in Amazonas, northwestern Peru. (a) Location of the department of Amazonas, elevation, and provincial boundaries. (b) Spatial distribution of the final cacao (Theobroma cacao L.) occurrence records retained for modeling after spatial filtering (n = 403). World country boundaries were obtained from Esri; departmental and provincial boundaries from the National Institute of Statistics and Informatics of Peru (INEI; https://ide.inei.gob.pe/#capas); and elevation data from the Shuttle Radar Topography Mission (SRTM) digital elevation model provided by the United States Geological Survey (USGS). Cacao occurrence records were compiled from GBIF, iNaturalist, field surveys, agricultural cooperatives, and published sources, as described in the Materials and methods. Maps were prepared by the authors in QGIS.

From the perspective of cacao cultivation and climatic suitability, low- to mid-elevation belts (≈200–1,200 m a.s.l.) are particularly relevant. These areas are mainly associated with the valleys of the Marañón, Utcubamba, Chinchipe, Nieva, Imaza, and Cenepa rivers, where agricultural and agroforestry systems are concentrated [34]. The climate encompasses warm-humid, warm-dry, and warm-temperate domains; temperatures range from temperate values at higher elevations to records approaching 40 °C in northern rainforest sectors. Annual precipitation exhibits marked spatial heterogeneity, ranging from less than 1,000 mm to more than 3,000 mm, primarily modulated by altitude and topography [39–41].

The biophysical landscape includes lowland forests, tropical dry forest, Andean forests and grasslands, and upper rainforest or yunga ecosystems (Atalaya et al., 2022), developed on predominantly acidic soils and exhibiting susceptibility to erosive processes on steep slopes [39]. Within this context, cacao has consolidated as a strategic crop for the rural economy and for productive restoration initiatives [19].

### Cacao presence data

Information on the spatial distribution of cacao in the department of Amazonas was compiled from multiple sources. Georeferenced presence records were obtained from the Global Biodiversity Information Facility (GBIF; https://www.gbif.org/) using the GBIF Occurrences plugin in QGIS 3.28 [19,24], and were complemented with records available on the iNaturalist platform (https://www.inaturalist.org). When some records lacked exact coordinates, these were manually determined through inspection of high-resolution satellite imagery in Google Earth Pro.

In addition, a proprietary database was developed that integrated presence records collected in the field using GPS, information provided by agricultural cooperatives involved in cacao production, and data derived from specialized scientific publications [42–44]. In total, 843 georeferenced records were compiled, including geographic coordinates (latitude and longitude) and the scientific name of the species.

To reduce spatial autocorrelation and prevent overestimation in environmental suitability models, a systematic data-cleaning procedure was applied [45]. This involved removing duplicates and retaining only one record per 250-m grid cell, corresponding to the spatial resolution of the environmental variables used. After this process, the final dataset comprised 403 unique cacao presence records.

### Environmental variables and preprocessing

A set of climatic, topographic, and edaphic variables was compiled as candidate environmental predictors for species distribution modeling (Table 1). Climatic predictors comprised the 19 bioclimatic variables available from WorldClim v2.1, derived from long-term climate records for the 1970–2000 period at a spatial resolution of approximately 1 km [46].

**Table 1:** Candidate environmental predictors considered in the biomod2 modeling framework.

Category	Variable	Symbol	Units
**Bioclimatic**	Annual mean temperature	Bio 1	°C
	Mean diurnal range	Bio 2	
	Isothermality (Bio2/Bio7) (* 100)	Bio 3	–
	Temperature seasonality (standard deviation *100)	Bio 4	–
	Max. temperature of the warmest month	Bio 5	°C
	Min. temperature of coldest month	Bio 6	
	Temperature annual range (Bio5-Bio6)	Bio 7	
	Mean temperature of wettest quarter	Bio 8	
	Mean temperature of driest quarter	Bio 9	
	Mean temperature of warmest quarter	Bio 10	
	Mean temperature of coldest quarter	Bio 11	
	Annual precipitation	Bio 12	mm
	Precipitation of wettest month	Bio 13	
	Precipitation of driest month	Bio 14	
	Precipitation seasonality	Bio 15	
	Precipitation of wettest quarter	Bio 16	
	Precipitation of driest quarter	Bio 17	
	Precipitation of warmest quarter	Bio 18	
	Precipitation of coldest quarter	Bio 19	
**Topographic**	Elevation above mean sea level	Altitude	m
	Terrain slope	Slope	°
	Terrain aspect	Aspect	
**Edaphic**	Topsoil sand fraction	Sand	%
	Topsoil silt fraction	Silt	
	Topsoil clay fraction	Clay	
	Organic carbon content	SOC	g/kg
	pH value (H_2_O)	phh2o	pH units
	Total nitrogen content	nitrogen	%
	The bulk density of the fine earth fraction	bdod	
	Volumetric fraction of coarse fragments	cfvo	

Topographic predictors were derived from the Shuttle Radar Topography Mission (SRTM) digital elevation model (DEM), obtained from the United States Geological Survey (USGS) at a spatial resolution of 30 m [47]. Elevation, slope, and aspect were derived from the DEM and included as candidate predictors.

Edaphic predictors were obtained from SoilGrids v2.0 (ISRIC–World Soil Information) at a spatial resolution of 250 m [48]. These variables included soil texture fractions (sand, silt, and clay), soil organic carbon, pH, total nitrogen, bulk density, and coarse-fragment content (Table 1).

To ensure spatial consistency among predictors, all environmental layers were projected to a common coordinate reference system, resampled, and aligned to a 250-m grid in QGIS 3.40 [49]. Continuous variables were resampled using bilinear interpolation, and all layers were clipped to the spatial extent of the study area before subsequent variable-selection and species distribution modeling procedures.

### Collinearity assessment and predictor selection

Multicollinearity among the candidate environmental predictors was assessed through a sequential procedure combining Pearson correlation analysis and the Variance Inflation Factor (VIF). First, pairwise Pearson correlation coefficients were calculated for the complete set of climatic, topographic, and edaphic variables (Fig 2a). To minimize redundancy among predictors, variables showing strong pairwise correlations (|r| ≥ 0.80) were progressively excluded [29,50]. This procedure resulted in a final subset of seven relatively independent predictors: coarse fragments (cfvo), temperature seasonality (bio04), silt content, bulk density (bdod), sand content, slope, and aspect.

**Fig 2:**
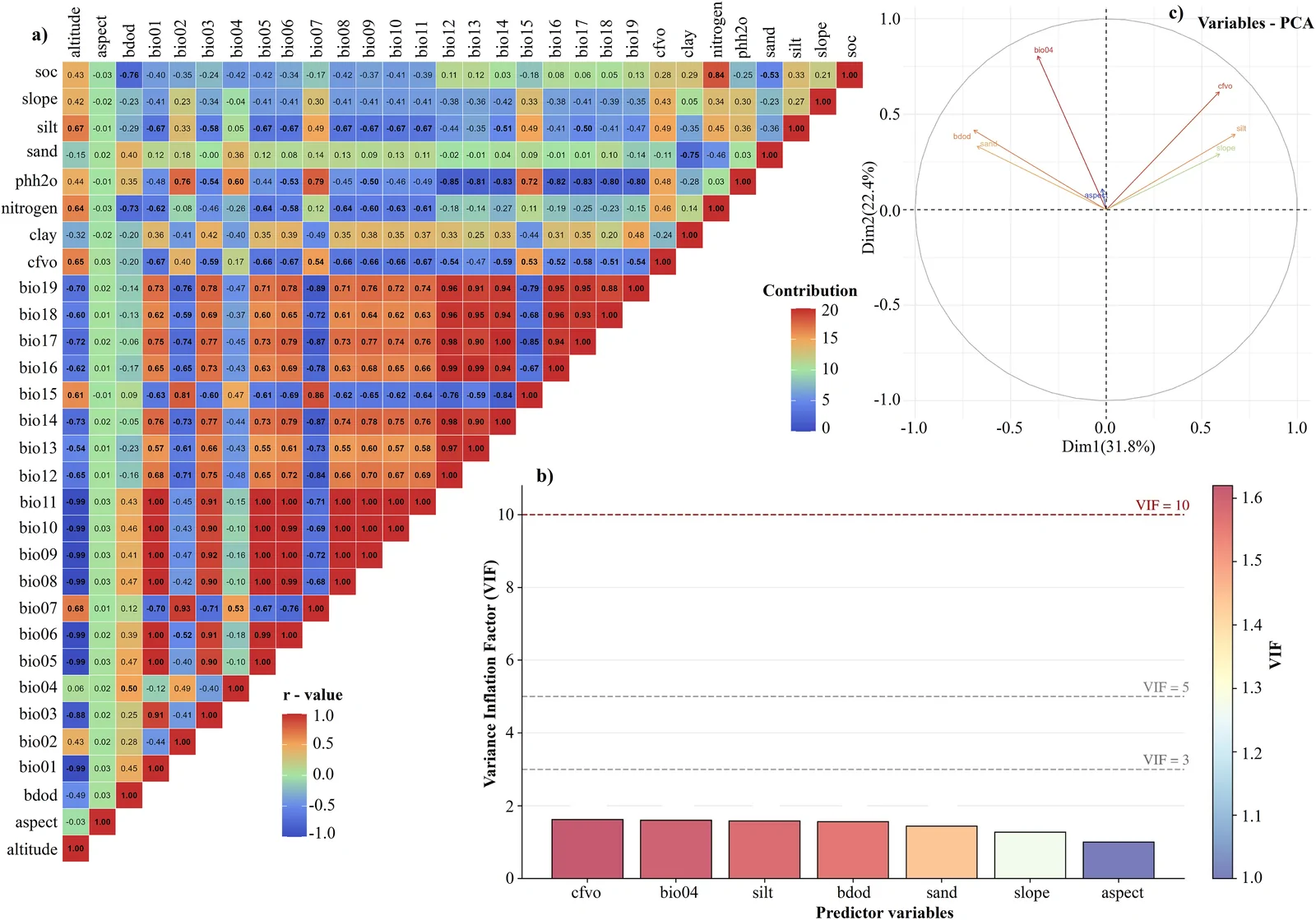
Collinearity assessment and predictor selection. (a) Pearson correlation matrix. (b) VIF values of the selected predictors. (c) PCA of the retained environmental variables**.**

Multicollinearity within the retained subset was subsequently evaluated using the VIF [51,52]. All selected predictors showed VIF values below 2 (Fig 2b), substantially lower than the commonly applied thresholds of 5 and 10 for identifying moderate and severe multicollinearity, respectively [53,54]. Therefore, all seven predictors were retained for subsequent species distribution modeling.

Finally, Principal Component Analysis (PCA) was applied to the retained predictors to characterize their multivariate relationships and visualize their association with the main environmental gradients [55]. The first two principal components explained 31.8% and 22.4% of the total variance, respectively, accounting for 54.2% of the combined variance (Fig 2c). The PCA was used as an exploratory visualization of the structure among the selected predictors rather than as a transformation of the variables used in the subsequent modeling.

### Construction and evaluation of SDMs

To estimate the current and future potential distribution of cacao under the SSP2–4.5 and SSP5–8.5 scenarios, an ensemble species distribution modeling (SDM) approach was applied using the biomod2 package (v. 4.2-6-2) [28] in R (v. 4.5.1) [56]. Biomod2 enables the standardized integration of multiple SDM algorithms, enhancing predictive robustness by reducing reliance on a single method and synthesizing consensus across models [57]. In this study, twelve algorithms implemented in biomod2 were evaluated [27,58]: artificial neural networks (ANN), flexible discriminant analysis (FDA), classification tree analysis (CTA), generalized additive models (GAM), generalized boosted models (GBM), generalized linear models (GLM), maximum entropy (MAXENT), an alternative maximum entropy implementation (MAXNET), multivariate adaptive regression splines (MARS), extreme gradient boosting (XGBOOST), random forest (RF), and species range envelope (SRE).

Because these algorithms require balanced presence–absence data [51,59,60], random pseudo-absences were generated in numbers equal to the available cacao presence records [31,61]. For calibration and evaluation, the dataset was partitioned into 80% for training and 20% for validation, following commonly applied SDM practices to minimize overfitting and ensure generalization [62]. Each algorithm was run for 10 repetitions, and k-fold cross-validation was applied to reduce variance associated with a single data split [50,63–65].

Model predictive performance was assessed using the Area Under the Curve (AUC) of the ROC curve and the True Skill Statistic (TSS). AUC values close to 1.0 indicate high discriminatory capacity and are operationally classified as excellent (0.9–1.0), good (0.8–0.9), poor (0.6–0.7), or failed (<0.6) [30,65,66]. TSS simultaneously accounts for omission and commission errors and corrects for chance agreement, being interpreted as excellent (>0.8), good (0.6–0.8), moderate (0.4–0.6), poor (0.2–0.4), or failed (<0.2), with TSS > 0.6 commonly accepted as an adequacy threshold [64,67]. Only individual models with AUC > 0.9 and TSS > 0.7 were retained for ensemble construction [30,68], SRE and MAXNET were excluded for not meeting performance criteria. Consequently, the final ensemble comprised 100 individual models (10 algorithms × 10 repetitions).

Prior to ensemble consolidation, predictor variable contributions were estimated. In addition, the build.clamping.mask option was used to spatially identify areas where projections involved extrapolation beyond the environmental domain represented by the training data [69]. The ensemble prediction was obtained as the mean probability across the selected models, producing a continuous suitability raster ranging from 0 to 1000, where higher values indicate greater suitability [62,70]. This raster was subsequently rescaled to 0–1 and reclassified into four suitability levels: unsuitable (0–0.2), low (0.2–0.4), moderate (0.4–0.6), and high (>0.6) [64]. This classification facilitates spatial interpretation and subsequent overlay analyses with exclusion and inclusion scenarios.

### Estimation of current suitable areas for cacao under exclusion and prioritization scenarios

To identify environmentally and legally feasible areas for cacao expansion in Amazonas, a sequential spatial filtering approach was applied using biophysical, infrastructure, conservation, forest-cover, and land-degradation criteria. The spatial distribution of the individual exclusion and prioritization criteria is shown in Fig 3. These layers were subsequently combined to construct three cumulative exclusion scenarios and one final prioritization scenario.

**Fig 3:**
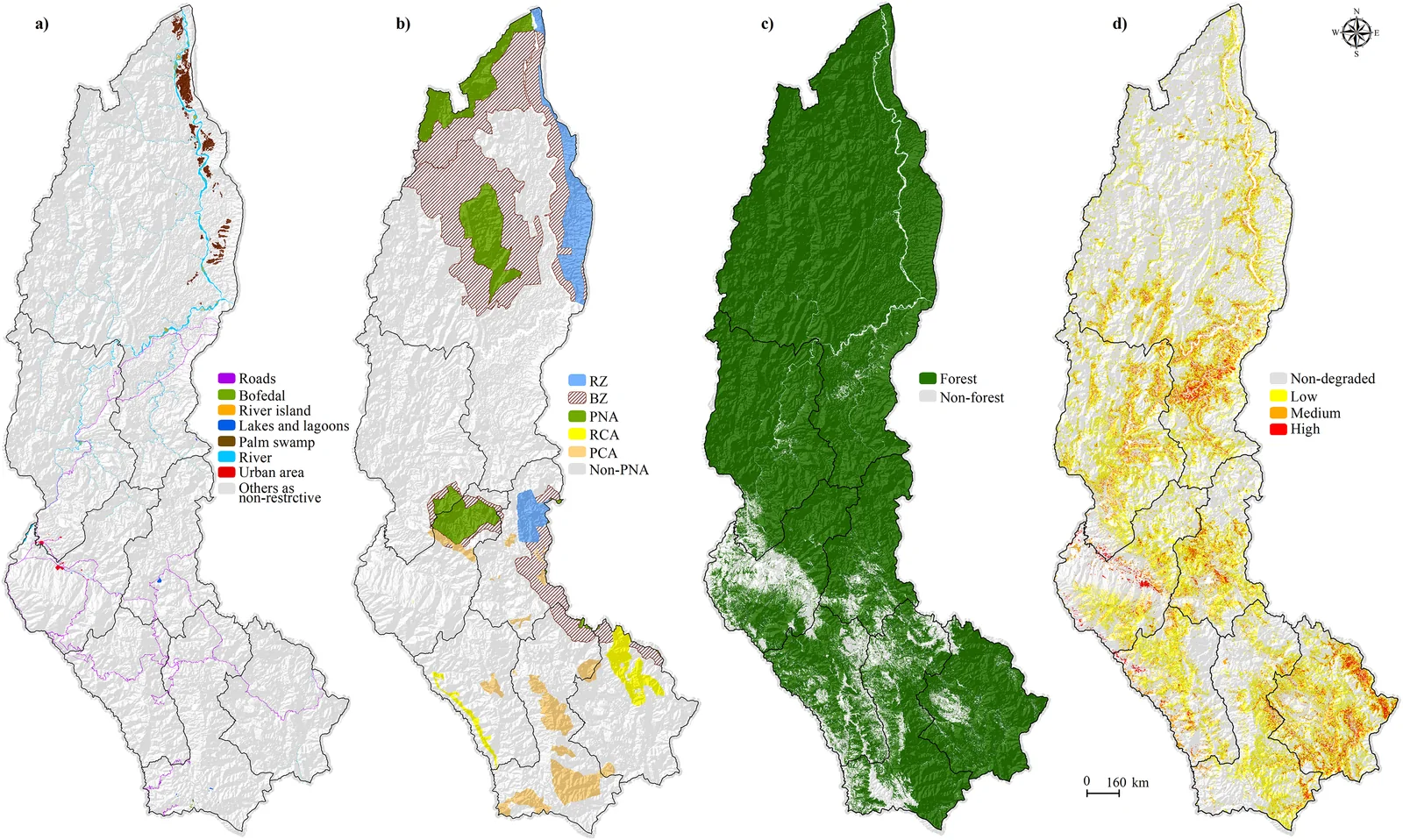
Criterios espaciales de exclusión y priorización de restauración utilizados para identificar áreas aptas para cacao en Amazonas, Perú. (a) Restricciones biofísicas y red vial. (b) Restricciones de conservación, incluidas las áreas protegidas y sus categorías asociadas. (c) Áreas de bosque y no bosque utilizadas para evaluar restricciones relacionadas con el Reglamento (UE) 2023/1115 (EUDR). (d) Clases de degradación de tierras utilizadas para identificar áreas prioritarias para restauración productiva. Los límites administrativos se obtuvieron del Instituto Nacional de Estadística e Informática del Perú (INEI); los datos de ecosistemas del Ministerio del Ambiente del Perú (MINAM); la red vial del Ministerio de Transportes y Comunicaciones del Perú (MTC); las áreas de conservación del Servicio Nacional de Áreas Naturales Protegidas por el Estado (SERNANP); la cobertura forestal del Joint Research Centre (JRC) de la Comisión Europea; y la degradación de tierras de MINAM. Los mapas fueron elaborados por los autores en QGIS.

#### Exclusion scenarios.

Three cumulative exclusion scenarios were established to progressively restrict areas considered suitable for cacao cultivation.

**Scenario 1. Biophysical and infrastructure restrictions.** Areas incompatible with agricultural establishment were excluded, including rivers, wetlands (bofedal), lakes and lagoons, palm swamps, and urban areas, based on the National Ecosystems Map [71]. Infrastructure restrictions associated with the national and departmental road network were also incorporated (Fig 3b).

**Scenario 2. Conservation restrictions.** Scenario 2 incorporated all restrictions from Scenario 1 and additionally excluded areas subject to conservation constraints, including Protected Natural Areas and their buffer zones, Regional Conservation Areas, Reserved Zones, and Private Conservation Areas, according to official SERNANP spatial information [72] (Fig 3b).

**Scenario 3. Forest-cover and EUDR-related restrictions.** Scenario 3 retained the restrictions applied in Scenario 2 and incorporated an additional forest-cover filter to account for requirements associated with deforestation-free production under Regulation (EU) 2023/1115 [32]. Forest-cover information from the Global Forest Cover Map 2020, Version 2, produced by the European Commission Joint Research Centre, was used for this purpose [73] (Fig 3c).

#### Prioritization scenario.

**Scenario 4. Prioritization of degraded lands.** Among areas remaining eligible after Scenario 3, highly degraded lands were identified as priority areas for productive restoration and potential expansion of cacao-based agroforestry systems **(**Fig 3d). Degradation information was obtained from the National Degraded Lands Map [74,75], which classifies land degradation into four severity categories (non-degraded, low, medium, and high) and identifies degradation processes such as vegetation-cover change (CVC), fragmentation, negative net primary productivity (NPP), forest loss, and combinations of these processes. This dataset has also been applied in previous restoration and land-suitability assessments [76,77].

In this study, only areas classified as highly degraded were considered for prioritization because they represent locations with greater restoration needs and potential for productive recovery. Their inclusion as priority areas was intended to guide cacao-based agroforestry expansion toward previously degraded landscapes while avoiding expansion into forested or legally restricted areas.

### Phytosanitary characterization and field validation

Modeling of cacao climatic suitability was complemented with field validation through active epidemiological surveillance, aimed at contrasting the spatial signal of climatic suitability with observational evidence of biotic pressure [78]. Under the assumption that bioclimatic conditions optimizing the physiological performance of Theobroma cacao—particularly relatively stable temperature regimes and high humidity—also favor key processes in the epidemiology, sporulation, and dispersal of its main pathogens, a partial overlap between climatically suitable areas and hotspots of frosty pod rot and witches’ broom was anticipated [79,80]. Similarly, warm–humid environments may increase the likelihood of co-occurrence with economically important phytophagous pests, including *Monalonion dissimulatum* and the *Antiteuchus spp*. complex [81,82].

Accordingly, a longitudinal database (2021–2024) was consolidated for the three provinces with the greatest relevance for cacao production in Amazonas: Utcubamba, Bagua, and Condorcanqui. Stratified sampling was applied to characterize phytosanitary pressure in sentinel units (n = 25 farms per district) and to estimate spatial prevalence (percentage of affected production units), following epidemiological criteria for sampling design and incidence estimation [83]. To capture damage intensity at higher resolution, 100 plants were evaluated per plot, recording incidence and severity at the plant level using standardized visual scales and best quantification practices to minimize assessment bias [84]. The biotic stress agents considered included the main fungal diseases of regional importance—frosty pod rot (*Moniliophthora roreri*) and witches’ broom (*M. perniciosa*)—as well as economically impactful entomological pests (*Monalonion dissimulatum* and *Antiteuchus spp.*) [79–82].

No specific institutional or governmental permits were required for the fieldwork because the phytosanitary assessments were conducted on privately owned cacao farms. Prior to entering each farm, the purpose of the field assessment was explained to the landowner, and verbal permission was obtained to access the property and conduct the phytosanitary evaluations

This approach enabled direct comparison between climatic suitability and empirical evidence of phytosanitary pressure, strengthening the ecological interpretation and territorial applicability of the models.

## Results

### Predictive performance of algorithms and base-model selection

The ten algorithms showed differences in predictive performance based on ROC–AUC and TSS values (Fig 4A–4C). ROC–AUC values were generally high across algorithms, although ANN showed the lowest values and the greatest difference relative to the highest-performing models (Fig 4a). A similar pattern was observed for TSS, with RF and XGBoost showing the highest values across model replicates (Fig 4b).

**Fig 4:**
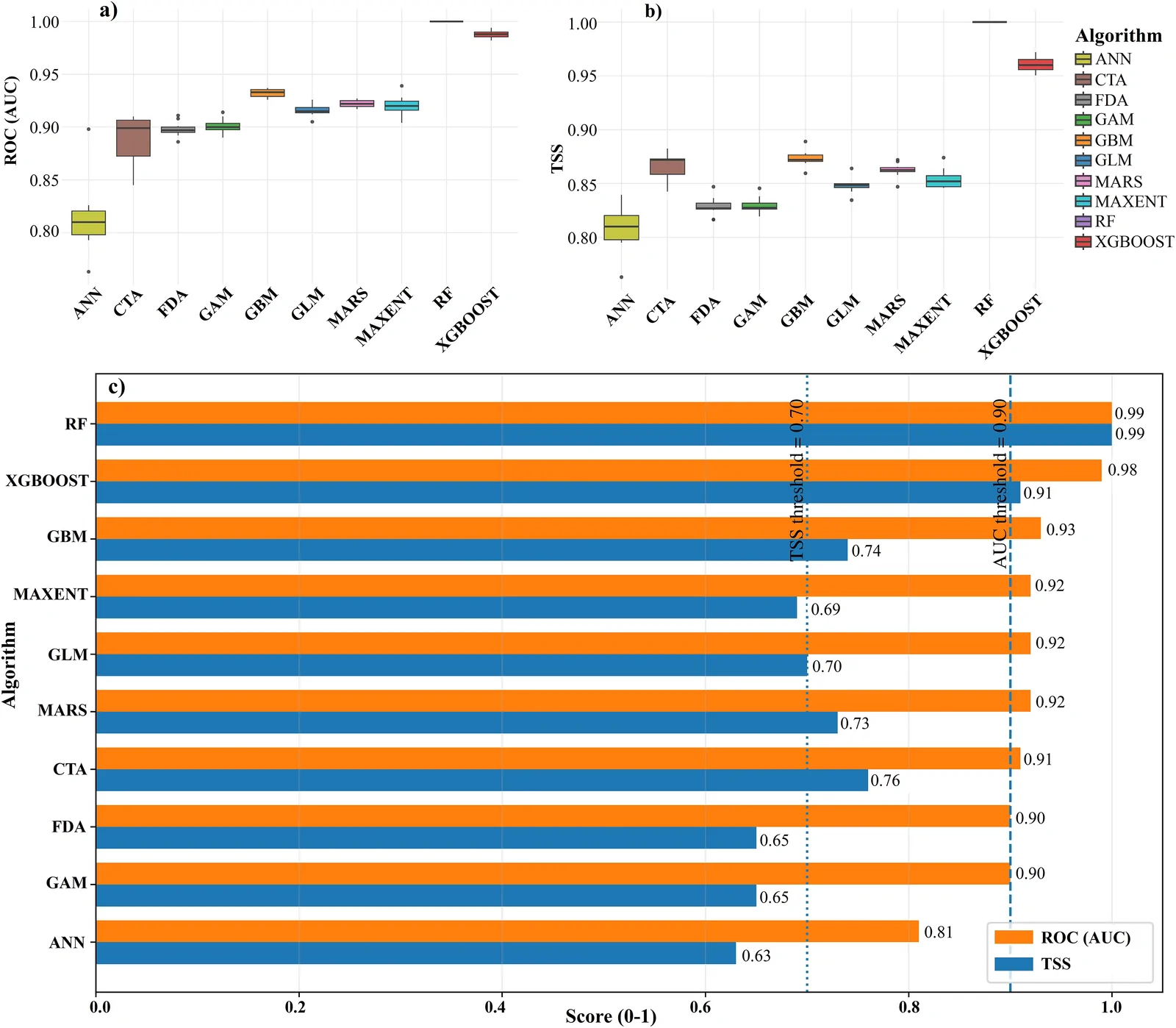
Predictive performance of algorithms used to model cacao suitability in Amazonas, Peru. (a) Distribution of ROC–AUC values across model replicates. (b) Distribution of TSS values across model replicates. (c) Mean ROC–AUC and TSS values for each algorithm; dashed lines indicate reference thresholds of AUC = 0.90 and TSS = 0.70.

Based on mean performance, RF achieved the highest scores for both ROC–AUC (0.99) and TSS (0.99), followed by XGBoost (AUC = 0.98; TSS = 0.91) (Fig 4c). GBM reached an AUC of 0.93 and a TSS of 0.74, whereas CTA, MARS, GLM, and MaxEnt showed AUC values ranging from 0.91 to 0.92 and TSS values between 0.69 and 0.76. FDA and GAM obtained AUC values of 0.90 and TSS values of 0.65, while ANN showed the lowest mean performance (AUC = 0.81; TSS = 0.63).

According to the combined ROC–AUC and TSS performance, RF ranked highest among the evaluated algorithms and was selected as the base model for subsequent spatial analyses.

### Environmental predictor importance, ensemble performance, and spatial suitability patterns

The relative importance of environmental predictors varied among algorithms, although temperature seasonality (bio04) consistently showed the highest contribution across all evaluated models (Fig 5a). Its importance ranged from 30.3% in RF to 87.9% in ANN, with particularly high contributions in CTA (68.0%), GBM (62.3%), MARS (61.8%), and XGBoost (62.1%). Among the remaining predictors, bulk density (bdod) showed comparatively high contributions in XGBoost (29.5%) and CTA (25.5%). Silt, sand, and slope reached their highest contributions in ANN, with values of 23.5%, 21.1%, and 19.1%, respectively, whereas aspect and coarse-fragment content (cfvo) generally showed lower contributions across algorithms.

**Fig 5:**
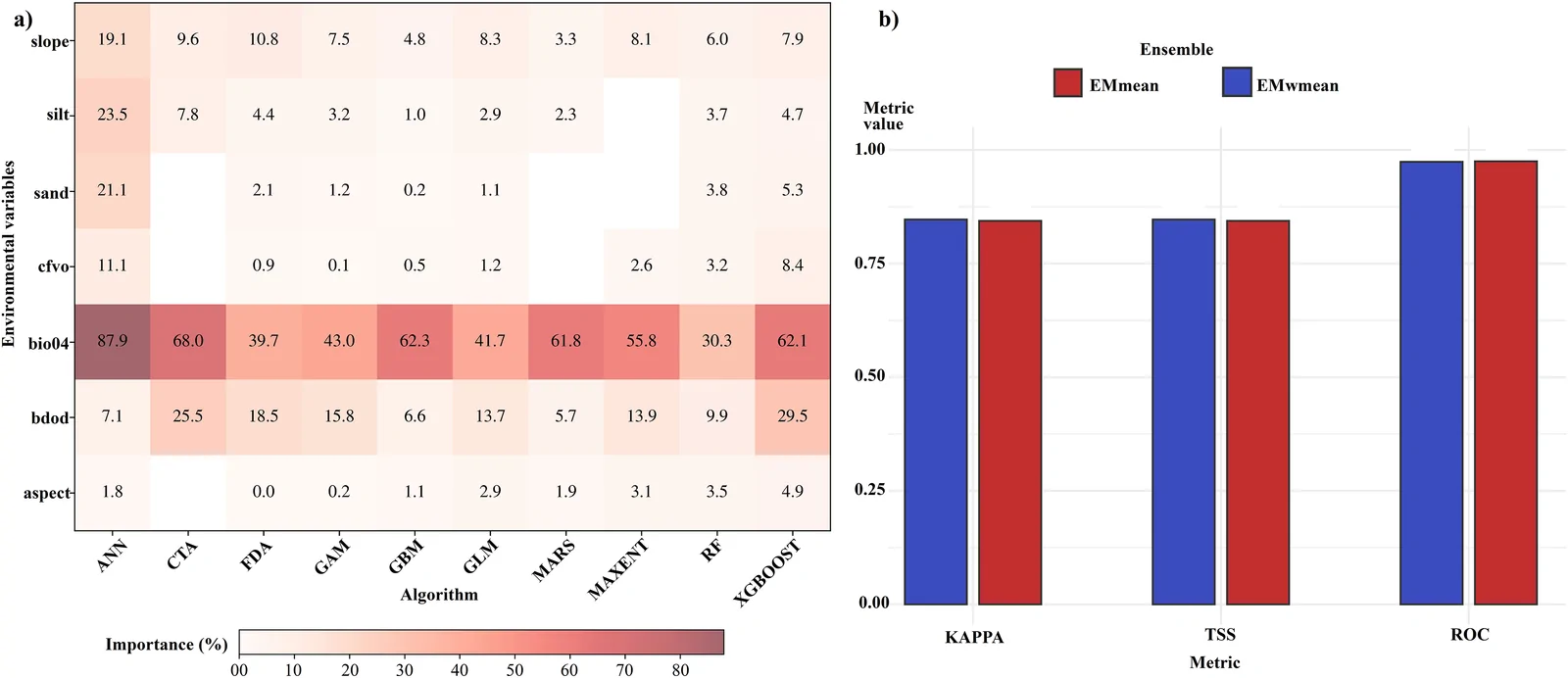
Environmental predictor importance and ensemble performance. (a) Relative importance (%) of environmental predictors across the evaluated algorithms. (b) Predictive performance of the simple-mean (EMmean) and weighted-mean (EMwmean) ensemble approaches based on Kappa, TSS, and ROC metrics.

The two ensemble approaches showed similarly high predictive performance across the three-evaluation metrics (Fig 5b). The simple-mean ensemble (EMmean) achieved Kappa and TSS values of 0.847 and a ROC value of 0.974, whereas the weighted-mean ensemble (EMwmean) achieved Kappa and TSS values of 0.844 and a ROC value of 0.975. Differences between both ensemble approaches were therefore small for all evaluated metrics.

Spatial predictions from the individual algorithms showed differences in the extent, continuity, and distribution of cacao suitability across Amazonas (Fig 6a–6e). RF, XGBoost, GBM, and MARS identified broad areas of moderate to high suitability, particularly in the central and southern sectors of the department, with additional suitable areas toward the north. RF, GBM, and MARS produced relatively continuous suitability patterns, whereas XGBoost showed a more fragmented spatial distribution in several sectors. CTA generated the most spatially restricted prediction, with suitable areas concentrated in more localized portions of the department. Despite these differences, the algorithms showed spatial agreement in several areas of moderate to high suitability, particularly in central and southern Amazonas.

**Fig 6:**
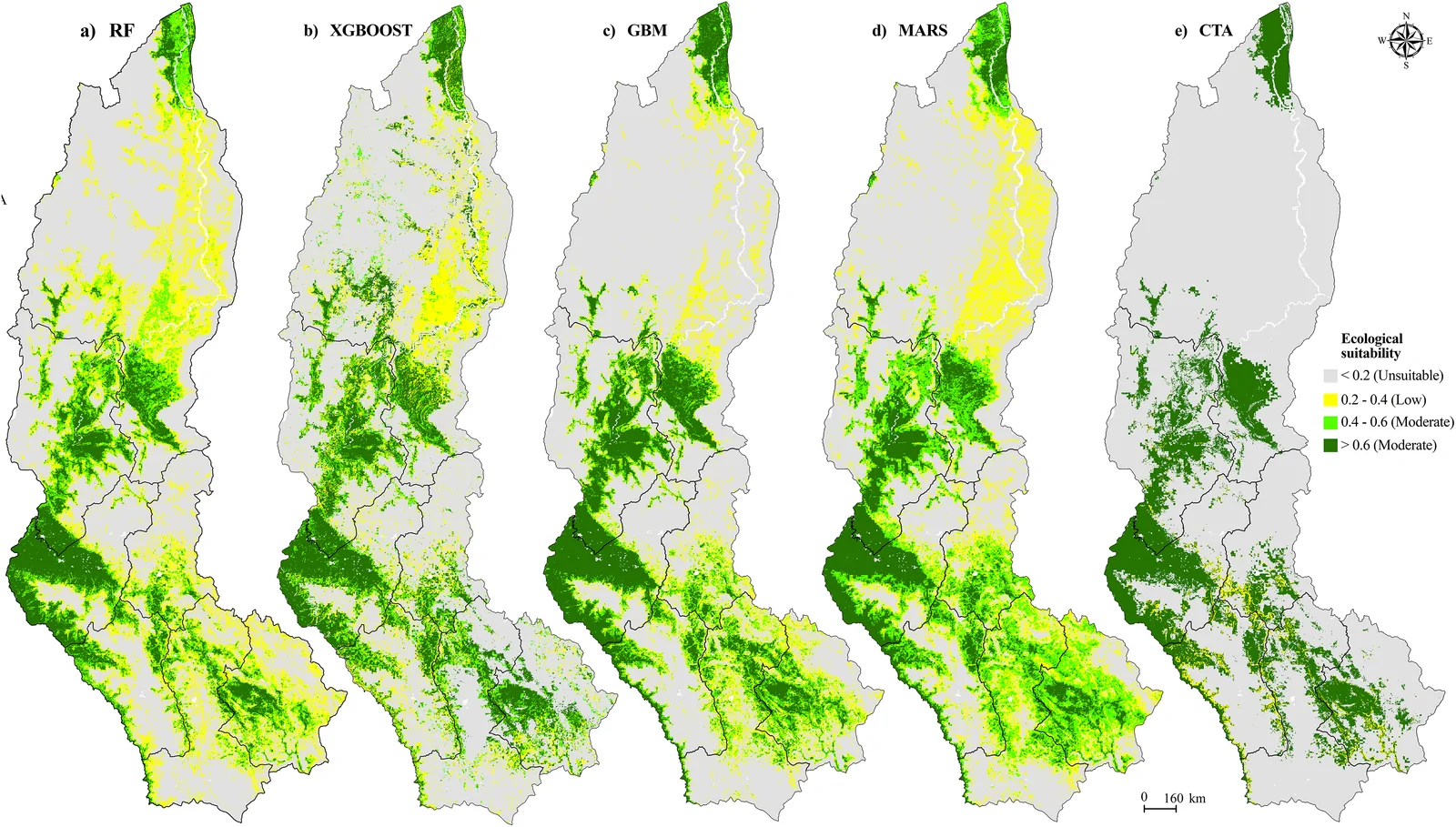
Spatial patterns of cacao suitability predicted by individual algorithms in Amazonas, Peru. Predictions generated by (a) RF, (b) XGBoost, (c) GBM, (d) MARS, and (e) CTA. Ecological suitability was classified as unsuitable (<0.2), low (0.2–0.4), moderate (0.4–0.6), and high (>0.6). Administrative boundaries were obtained from the National Institute of Statistics and Informatics of Peru (INEI). Suitability maps were generated by the authors using the environmental predictors and modeling procedures described in the Materials and methods.

### Geographic distribution of current cacao suitability under exclusion and restoration-prioritization scenarios

Under current conditions and before applying spatial restrictions, cacao suitability showed a heterogeneous distribution across Amazonas (Fig 7a). Unsuitable areas (S1) (<0.2) represented 57.4% of the department (23,960.86 km^2^), followed by low suitability (S2) (0.2–0.4) with 18.9% (7,866.17 km^2^), moderate suitability (S3) (0.4–0.6) with 8.9% (3,705.00 km^2^), and high suitability (S4) (>0.6) with 14.8% (6,184.86 km^2^). High-suitability areas were mainly distributed across the central and southern portions of Amazonas, with additional areas in the northern sector.

**Fig 7:**
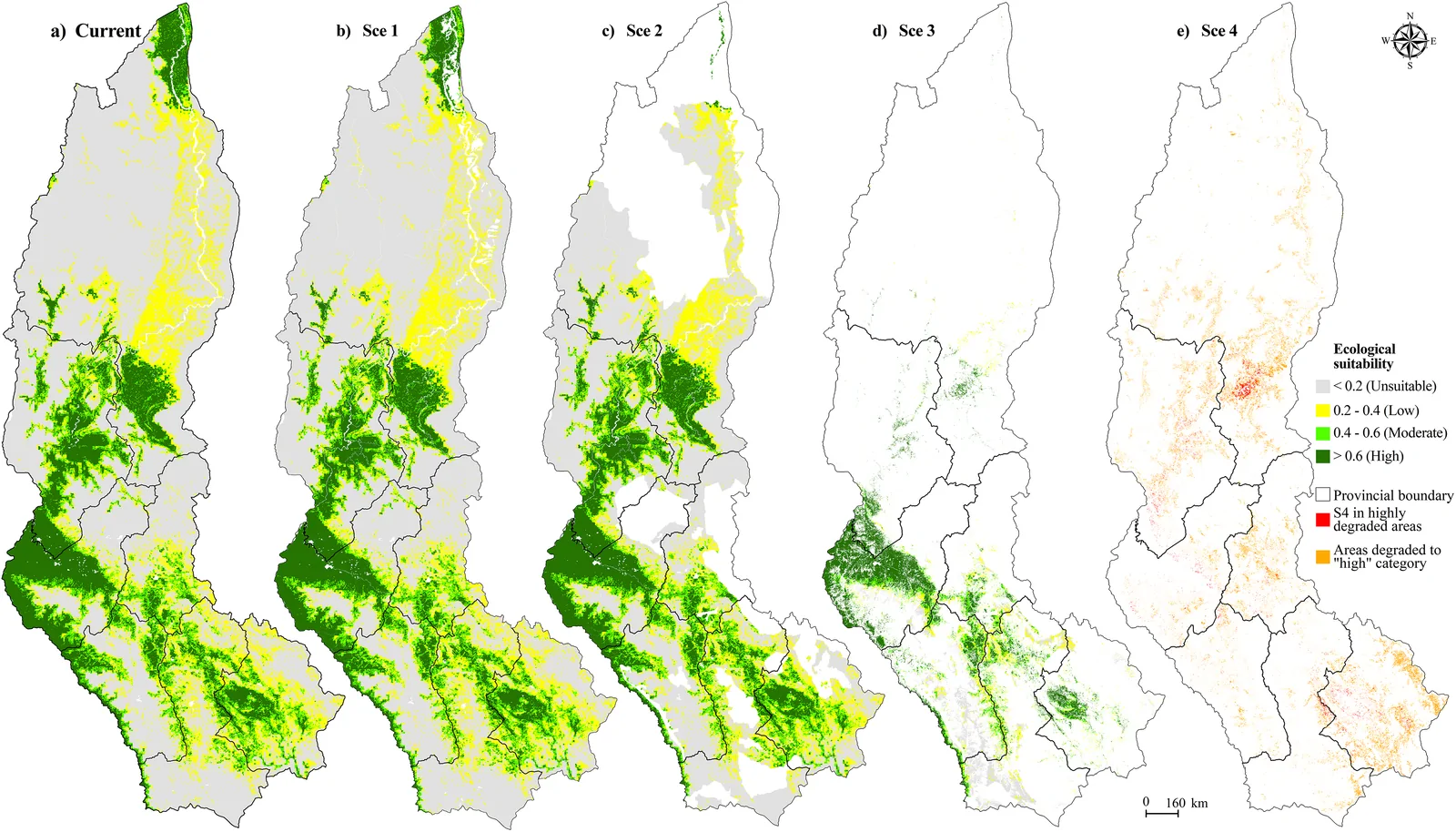
Current cacao suitability under exclusion and restoration-prioritization scenarios in Amazonas, Peru. (a) Current environmental suitability without spatial restrictions. (b) Scenario 1 after biophysical and infrastructure exclusions. (c) Scenario 2 after the addition of conservation restrictions. (d) Scenario 3 after the addition of forest-cover restrictions associated with Regulation (EU) 2023/1115 (EUDR). (e) Scenario 4 showing highly degraded lands overlapping the high-suitability areas remaining after Scenario 3. Administrative boundaries were obtained from the National Institute of Statistics and Informatics of Peru (INEI); spatial restriction and degradation datasets were obtained from the official sources described in the Materials and methods. Maps were generated by the authors.

At the provincial level, Utcubamba and Bagua contained the largest high-suitability areas, with 1,635.36 and 1,502.05 km^2^, respectively, followed by Condorcanqui with 1,473.34 km^2^ (Table 2). In contrast, Bongará contained the smallest high-suitability area (196.96 km^2^). Condorcanqui also accounted for the largest extent classified as unsuitable (12,621.99 km^2^), whereas Chachapoyas contained 2,371.74 km^2^ in this class.

**Table 2:** Area (km^2^) and percentage (%) of cacao suitability classes by province in Amazonas, Peru.

Province	S1 km^2^ (%)	S2 km^2^ (%)	S3 km^2^ (%)	S4 km^2^ (%)
**Bagua**	2,721.13 (6.5)	795.72 (1.9)	801.74 (1.9)	1,502.05 (3.6)
**Bongará**	1,767.98 (4.2)	683.22 (1.6)	366.64 (0.9)	196.96 (0.5)
**Chachapoyas**	2,371.74 (5.7)	1,237.82 (3.0)	529.39 (1.3)	355.47 (0.9)
**Condorcanqui**	12,621.99 (30.3)	3,001.96 (7.2)	536.32 (1.3)	1,473.34 (3.5)
**Luya**	1,553.61 (3.7)	511.53 (1.2)	505.53 (1.2)	525.24 (1.3)
**Rodríguez de Mendoza**	1,421.15 (3.4)	1,220.30 (2.9)	574.05 (1.4)	496.44 (1.2)
**Utcubamba**	1,503.25 (3.6)	415.63 (1.0)	391.34 (0.9)	1,635.36 (3.9)
**Total**	23,960.86 (57.4)	7,866.17 (18.9)	3,705.00 (8.9)	6,184.86 (14.8)

Application of the cumulative exclusion criteria progressively reduced the spatial extent available within each suitability class (Fig 7b–7d; Table 3). Under Scenario 1, which incorporated biophysical and infrastructure restrictions, the high-suitability area decreased from 6,184.86 km² (14.8%) to 5,932.98 km² (14.2%) (Fig 7c). After incorporating conservation restrictions in Scenario 2, the remaining high-suitability area decreased to 5,428.33 km² (13.0%) (Fig 7c).

**Table 3:** Area (km^2^) and percentage (%) of cacao suitability classes under cumulative exclusion and restoration-prioritization scenarios in Amazonas, Peru.

Modeling scenarios	S1 km^2^	S1% (Dif)	S2 km²	S2% (Dif)	S3 km^2^	S3% (Dif)	S4 km^2^	S4% (Dif)
Scenario 1	23,813.24	57.1 (−0.4)	7,712.70	18.5 (−0.4)	3,642.52	8.7 (−0.2)	5,932.98	14.2 (−0.6)
Scenario 2	14,550.74	34.9 (−22.6)	5,969.42	14.3 (−4.6)	3,264.46	7.8 (−1.1)	5,428.33	13.0 (−1.8)
Scenario 3	961.57	2.3 (−55.1)	695.90	1.7 (−17.2)	659.81	1.6 (−7.3)	1,918.35	4.6 (−10.2)
Scenario 4	—	—	—	—	—	—	46.28	0.1

*Note: Δ represents the percentage-point difference relative to the current suitability distribution. Scenarios 1–3 were applied cumulatively. Scenario 4 reports only highly degraded lands overlapping the high-suitability areas remaaining after Scenario 3.*

The largest reduction occurred after application of the forest-cover restriction in Scenario 3, associated with the EUDR criterion, which reduced the remaining high-suitability area to 1,918.35 km² (4.6% of the departmental area) (Fig 7d). This represented a reduction of 4,266.51 km^2^ relative to the initial high-suitability area.

Finally, Scenario 4 identified highly degraded lands overlapping the high-suitability areas remaining after the previous exclusions (Fig 7e). This intersection comprised 46.28 km^2^, equivalent to approximately 0.1% of the departmental area and 2.4% of the high-suitability area remaining under Scenario 3. These areas were subsequently considered priority locations for productive restoration with cacao-based agroforestry systems.

### Phytosanitary dynamics and incidence across cacao-producing districts of Amazonas

Longitudinal phytosanitary monitoring conducted between 2021 and 2024 across 12 cacao-producing districts of the Amazonas department, Peru, revealed marked differences in the prevalence, incidence, and temporal behavior of the main diseases and insect pests affecting cacao. The evaluated agents included frosty pod rot (*Moniliophthora roreri*), witches’ broom (*Moniliophthora perniciosa*), *Monalonion dissimulatum*, and *Antiteuchus spp*. Their prevalence showed distinct spatial and temporal patterns among districts, with fungal diseases generally reaching higher and more persistent values than insect pests (Fig 8a–8d).

**Fig 8:**
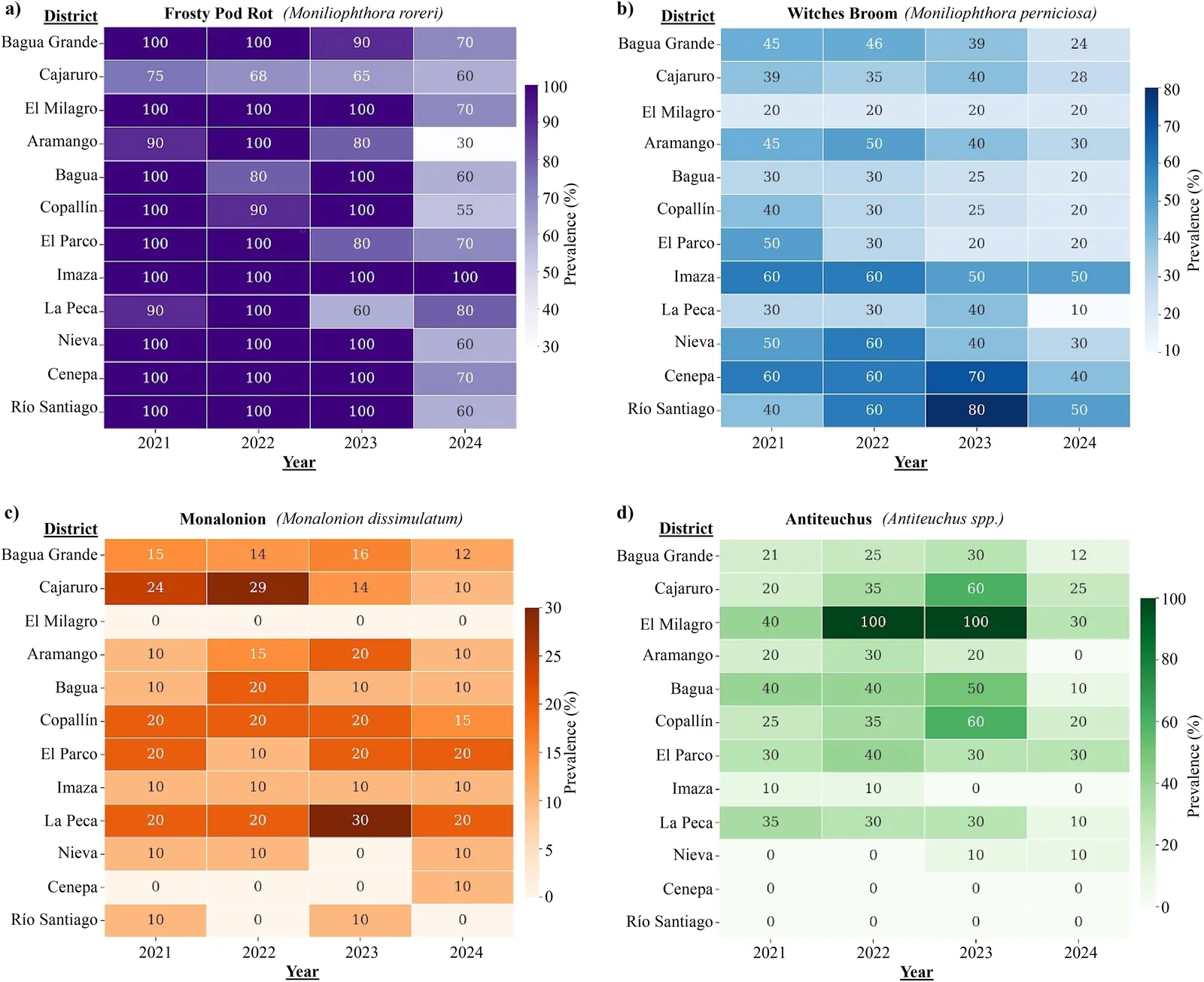
Spatio-temporal prevalence of cacao pests and diseases across districts of Amazonas, Peru (2021–2024). Prevalence (%) of (a) frosty pod rot (*Moniliophthora roreri*), (b) witches’ broom (*Moniliophthora perniciosa*), (c) *Monalonion dissimulatum*, and (d) *Antiteuchus spp*.

Frosty pod rot showed the highest prevalence throughout the monitoring period, with values between 60% and 100% in most districts during 2021–2023 (Fig 8a). Although prevalence decreased in several districts in 2024, high values persisted in specific locations, particularly Imaza, where 100% prevalence was recorded. Witches’ broom showed a more heterogeneous distribution, with prevalence ranging from 10% to 80% and the highest values occurring in Cenepa and Río Santiago during 2023 (Fig 8b). In comparison, *M. dissimulatum* generally showed lower prevalence, whereas *Antiteuchus spp*. exhibited strong interdistrict and interannual variation, including localized peaks of 100% in El Milagro during 2022 and 2023 (Fig 8c–8d).

### Prevalence and incidence of fungal pathogens

Frosty pod rot was the dominant fungal disease across the evaluated districts, both in prevalence and direct damage. In addition to the consistently high prevalence observed during 2021–2023 (Fig 8a), incidence/direct damage also reached high levels in several districts (Fig 9a). Nieva, Cenepa, and Río Santiago recorded values of 100% during 2021–2023, followed by reductions to 60%, 70%, and 60%, respectively, in 2024. Other districts also showed substantial interannual variation; for example, Aramango reached 90% incidence/direct damage in 2023 but decreased to 40% in 2024. These results show that the reduction observed toward the end of the monitoring period was not spatially uniform and that comparatively high levels remained in several districts of Amazonas.

**Fig 9:**
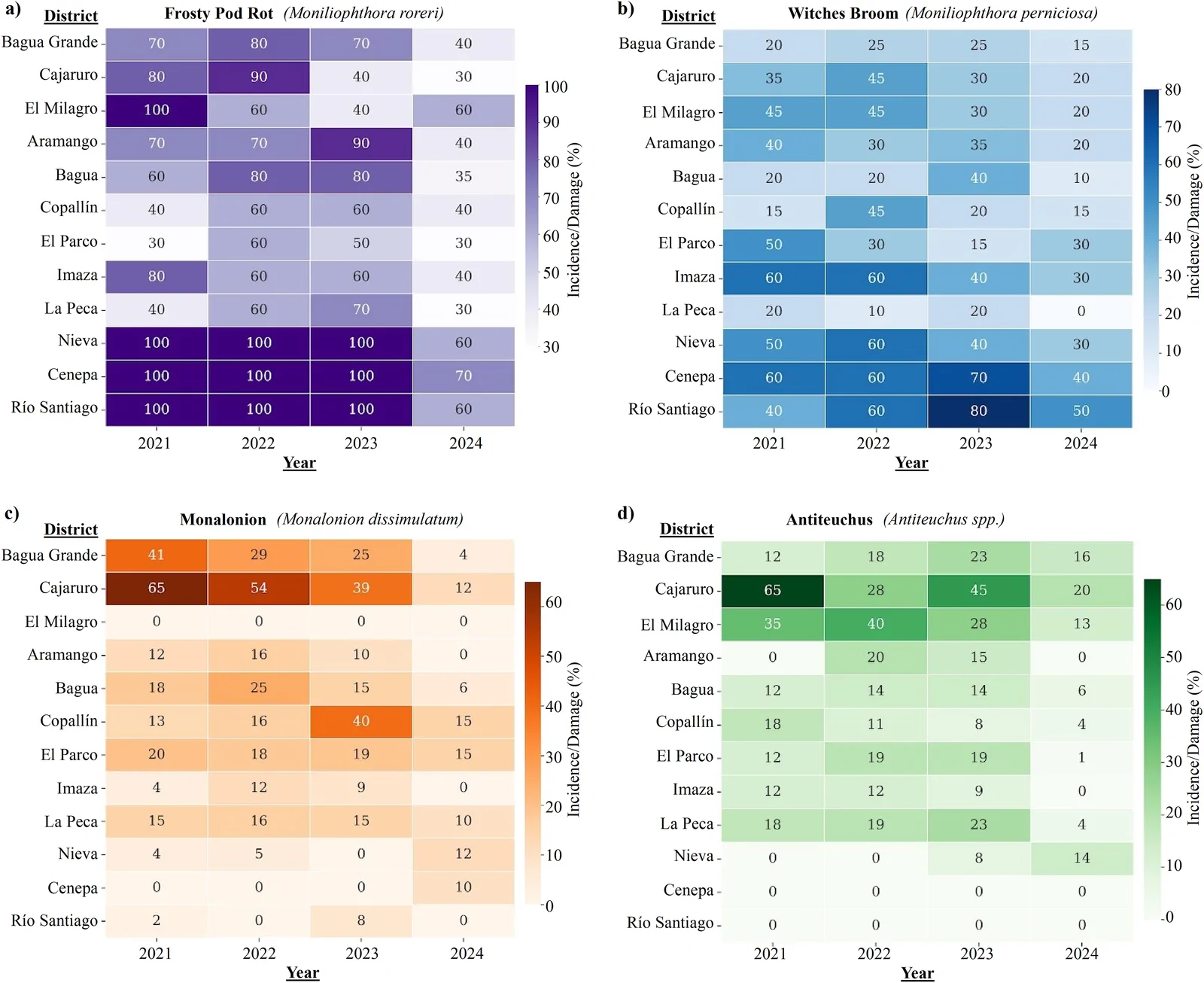
Incidence and direct damage caused by cacao pests and diseases across districts of Amazonas, Peru (2021–2024). Incidence/direct damage (%) associated with (a) frosty pod rot (*Moniliophthora roreri*), (b) witches’ broom (*Moniliophthora perniciosa*), (c) *Monalonion dissimulatum*, and (d) *Antiteuchus spp*.

Witches’ broom showed lower incidence/direct damage than frosty pod rot but maintained recurrent occurrence in several districts (Fig 9b). The highest values were observed in Río Santiago, where incidence/direct damage increased from 40% in 2021 to 80% in 2023 before declining to 50% in 2024. Cenepa similarly increased from 60% in 2021–2022 to 70% in 2023 and subsequently decreased to 40% in 2024. Imaza and Nieva also maintained relatively high values during the first years of monitoring. Overall, fungal disease pressure differed among districts, with frosty pod rot showing broader and consistently higher levels and witches’ broom displaying a more spatially heterogeneous pattern.

### Harmful entomofauna: Monalonion and Antiteuchus

The insect pests showed more localized and temporally variable patterns than the fungal pathogens. Monalonion dissimulatum generally presented low to moderate prevalence across the monitored districts (Fig 8c), with values ranging from 0% to 30%. The highest prevalence was recorded in La Peca in 2023 (30%), while several districts showed no recorded occurrence during one or more years. However, incidence/direct damage showed more pronounced localized values (Fig 9c), particularly in Cajaruro, where damage reached 65% in 2021 and 54% in 2022 before declining to 12% in 2024. Copallín also reached 40% in 2023, whereas values in most other districts remained comparatively low.

*Antiteuchus spp.* showed the greatest interdistrict variability among the insect pests evaluated (Fig 8d). El Milagro recorded prevalence values of 100% in both 2022 and 2023, while Cajaruro and Copallín reached 60% in 2023. In contrast, Cenepa and Río Santiago recorded no prevalence throughout the monitoring period. Patterns of incidence/direct damage were similarly heterogeneous (Fig 9d), with Cajaruro reaching the highest value (65%) in 2021, followed by 45% in 2023. Most districts showed declining damage toward 2024, and several recorded values close to or equal to zero.

### Longitudinal dynamics and regional trends

The comparison between 2021 and 2024 showed an overall decline in phytosanitary prevalence across most district–agent combinations, although the magnitude and direction of change differed among the four biotic agents (Fig 10a–10d). Antiteuchus spp. showed the most heterogeneous district-level response (Fig 10a), with decreases in several districts, including Bagua, La Peca, Aramango, El Milagro, and Imaza, while increases were observed in Cajaruro and Nieva. In contrast, *Monalonion dissimulatum* showed predominantly stable or declining prevalence between 2021 and 2024 (Fig 10b), although Cenepa recorded an increase of 10 percentage points.

**Fig 10:**
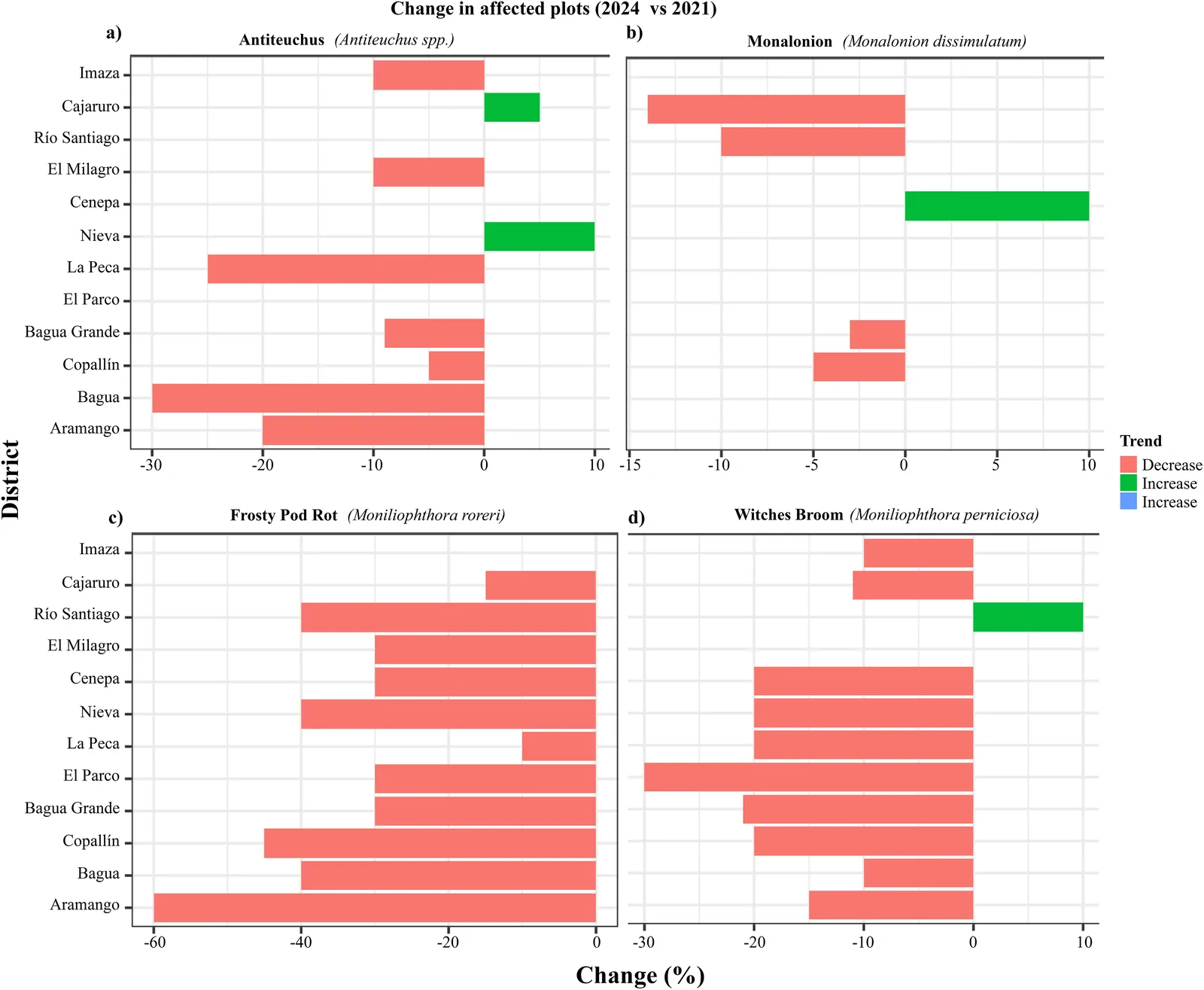
Change in phytosanitary prevalence between 2021 and 2024 across cacao-producing districts of Amazonas, Peru. Changes in prevalence are shown for (a) *Antiteuchus spp*., (b) *Monalonion dissimulatum*, (c) frosty pod rot (*Moniliophthora roreri*), and (d) witches’ broom (*Moniliophthora perniciosa*). Values are expressed as percentage-point differences between 2024 and 2021; positive values indicate increases and negative values indicate decreases.

Frosty pod rot (*Moniliophthora roreri*) showed the most consistent decline among districts (Fig 10c). The largest reduction occurred in Aramango, where prevalence decreased by 60 percentage points, while substantial decreases were also recorded in Bagua, Copallín, Nieva, Cenepa, Río Santiago, El Milagro, and Bagua Grande. Witches’ broom (*Moniliophthora perniciosa*) similarly declined in most districts (Fig 10d), with the largest reductions observed in El Parco, Bagua Grande, Copallín, and Nieva. Río Santiago was the main exception, showing an increase of 10 percentage points between 2021 and 2024.

At the regional scale, annual mean prevalence showed distinct temporal trajectories among the four phytosanitary agents (Fig 11a–11d). Antiteuchus spp. increased from 20.1% in 2021 to 28.8% in 2022 and reached its highest regional mean in 2023 (32.5%), followed by a marked decline to 12.3% in 2024 (Fig 11a). In contrast, M. dissimulatum remained comparatively stable between 2021 and 2023, with regional means ranging from approximately 12.3% to 12.5%, before decreasing to 10.6% in 2024 (Fig 11b).

**Fig 11:**
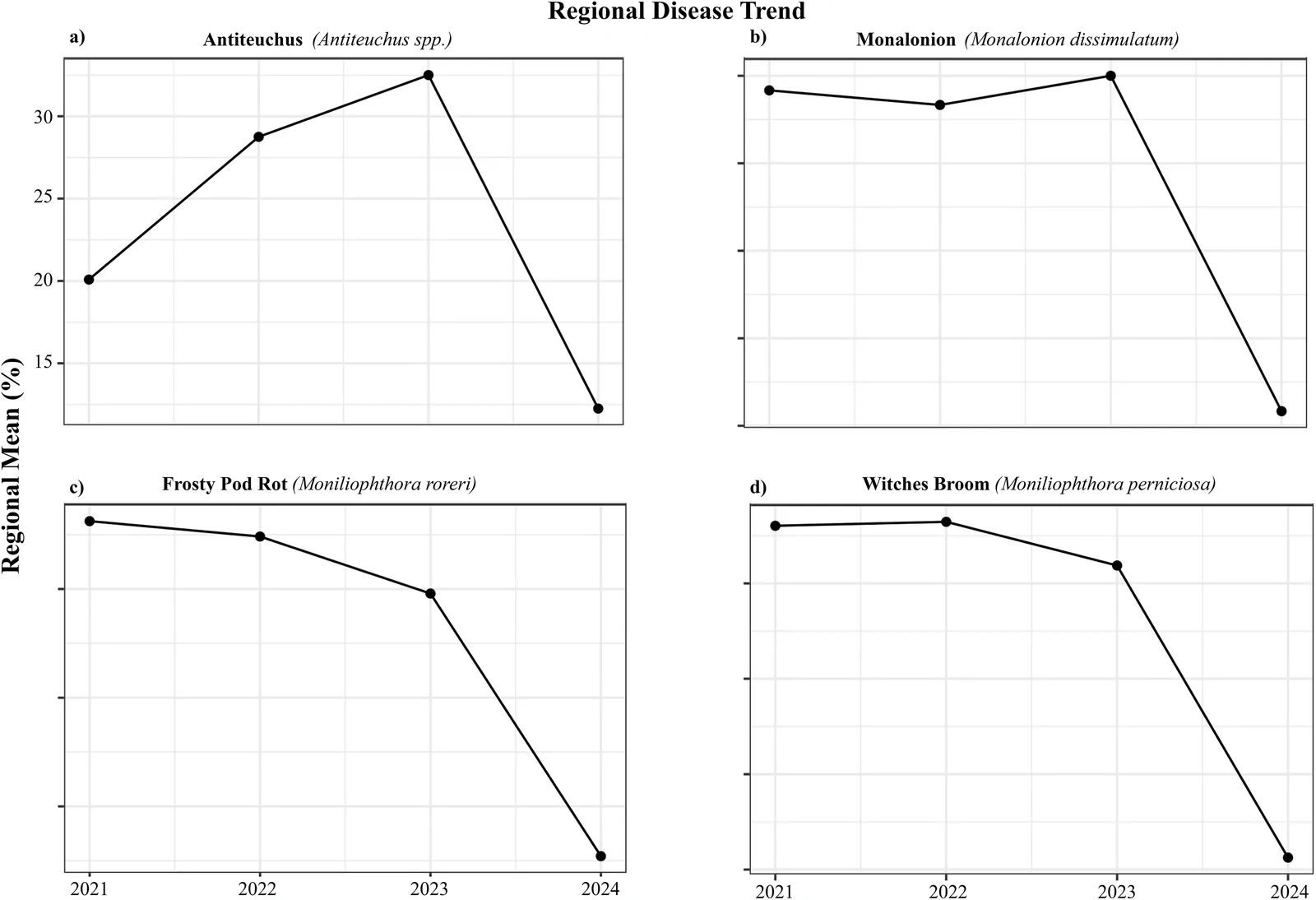
Regional temporal trends in cacao pest and disease prevalence in Amazonas, Peru (2021–2024). Annual regional mean prevalence (%) of *Antiteuchus spp*., *Monalonion dissimulatum*, frosty pod rot (*Moniliophthora roreri*), and witches’ broom (*Moniliophthora perniciosa*).

Frosty pod rot maintained the highest regional prevalence throughout the monitoring period (Fig 11c), decreasing from 96.3% in 2021 to 94.8% in 2022 and 89.6% in 2023, followed by a more pronounced reduction to 65.4% in 2024. Witches’ broom showed a comparatively stable regional prevalence during 2021–2023, with values of 42.4%, 42.6%, and 40.8%, respectively, before declining to 28.5% in 2024 (Fig 11d). Overall, despite differences in their temporal trajectories, all four phytosanitary agents recorded lower regional mean prevalence in 2024 than in the preceding years.

## Discussion

The use of ensemble-based species distribution models (SDMs) has become a robust methodological approach for assessing the environmental suitability of perennial crops in tropical regions characterized by high climatic and topographic heterogeneity [26,59,85]. In this study, applying an ensemble framework through biomod2 enabled the integration of multiple algorithms and reduced uncertainty associated with reliance on a single predictive method—an advantage widely documented in recent assessments of tropical crops and forest species [51,57,63].

The marked superiority of Random Forest and XGBoost over parametric and semi-parametric algorithms is consistent with studies showing that decision-tree–based methods have greater capacity to capture nonlinear relationships and complex interaction effects among environmental predictors [58,68]. In particular, Random Forest has consistently demonstrated high performance in agricultural suitability and ecological niche modeling under abrupt environmental gradients, owing to its tolerance to noise and residual collinearity among variables [31,65]. By contrast, models such as GLM and GAM, while useful for ecological inference, tend to underestimate suitability in complex landscapes by assuming simpler functional relationships among predictors [64,67].

The improvement observed in consensus model performance metrics relative to individual models reinforces evidence that ensembles not only increase overall accuracy but also stabilize the spatial signal of predictions, reducing local artifacts associated with specific algorithms [27,28]. This stability is particularly relevant in regions such as Amazonas, where small topographic variations generate significant microclimatic contrasts that can amplify uncertainty in individual models [29,37].

From an ecophysiological perspective, the dominance of temperature seasonality (Bio04) as the main predictor of climatic suitability aligns with studies highlighting cacao’s sensitivity to annual thermal variability rather than to mean temperature values [2,18]. Relative thermal stability favors key processes such as continuous flowering, fruit development, and reduced physiological stress—critical elements for sustained productivity [3,86]. Similar results have been reported in suitability assessments for cacao and coffee in Latin America, where thermal seasonality emerges as a structural limiting factor under current conditions [87,88].

The secondary contribution of edaphic variables, particularly soil bulk density (bdod), suggests that while climate defines the general suitability framework, soil physical properties modulate productive viability at local scales. This pattern is well documented in studies integrating SDMs with soil variables, indicating that compacted or low-porosity soils can constrain root development and water-use efficiency even in climatically favorable environments [38,48]. Conversely, the low relative contribution of topographic variables such as slope and aspect supports the view that, at the regional scale analyzed, these factors act mainly as secondary modulators of local climate [19,37].

The proportion of high climatic suitability identified for Amazonas (≈14.8%) is comparable to estimates from previous studies in Peru and the western Amazon, although slightly lower than those obtained using less restrictive approaches to variable selection and validation [18,19]. This difference suggests that the conservative approach adopted here prioritizes predictive robustness over maximizing potential area—an approach recommended for territorial planning and public policy applications [51,85].

Incorporating exclusion scenarios reveals that a substantial fraction of climatically suitable areas overlaps with territories subject to legal and regulatory constraints, particularly those related to conservation and the European Union Deforestation-Free Products Regulation (EUDR). The drastic reduction of class S4 under the EUDR scenario is consistent with recent assessments indicating that agricultural expansion potential in the Amazon is increasingly constrained by international regulatory frameworks rather than by biophysical limitations [7,32,73]. This result underscores the need to integrate regulatory eligibility criteria from the earliest stages of productive planning, as proposed in recent studies on sustainable cacao supply chains [8,16].

The inclusion scenario for degraded areas, although spatially limited, is strategically relevant as it identifies opportunities for productive restoration through cacao-based agroforestry systems. This approach is consistent with global frameworks promoting the reconversion of degraded lands as a pathway to reconcile agricultural production, ecological restoration, and deforestation mitigation [74,76]. Recent studies in the Peruvian Amazon suggest that, although scarce, these areas can generate disproportionately high environmental and socioeconomic benefits when properly prioritized [9,19].

Phytosanitary validation provides empirical evidence that areas of higher climatic suitability for cacao coincide with increased pressure from pathogens and pests, confirming that climatic niches optimal for the crop also favor the epidemiology of agents such as Moniliophthora roreri and M. perniciosa [23,79,80]. This overlap has been previously reported in phytosanitary risk studies under current climatic conditions, where host climatic suitability correlates positively with pathogen establishment and persistence [21,24].

The persistence of frosty pod rot as the main phytosanitary constraint, even under a declining regional trend, is consistent with recent reviews identifying it as the most limiting cacao disease in Latin America, particularly in warm–humid environments [22,25]. Complementarily, the focal behavior of pests such as Monalonion dissimulatum and Antiteuchus spp. aligns with studies highlighting their strong dependence on microclimatic conditions and local agronomic management [81,82].

Overall, the results confirm that high climatic suitability does not necessarily imply lower sanitary risk; rather, it may amplify biotic pressure if not accompanied by preventive integrated management strategies. This conclusion reinforces recent proposals advocating the integration of climatic, productive, and phytosanitary information within a unified agroecological planning framework, particularly in contexts of regulated expansion and increasing sustainability requirements [7,25,78].

## Conclusions

This study identified marked spatial differences in current cacao suitability across Amazonas, Peru. High-suitability areas represented 14.8% of the department (6,184.86 km^2^) and were mainly concentrated in Utcubamba (1,635.36 km^2^), Bagua (1,502.05 km^2^), and Condorcanqui (1,473.34 km^2^). Temperature seasonality was the most influential environmental predictor, while ensemble modeling produced consistently high predictive performance. The progressive incorporation of territorial restrictions substantially reduced the area available for cacao cultivation, particularly after applying forest-cover criteria associated with EUDR requirements, under which high-suitability areas decreased to 4.6% of the departmental territory (1,918.35 km^2^).

Within the areas remaining suitable after these restrictions, only 46.28 km^2^ of highly degraded land were identified as priority zones for cacao-based productive restoration. Phytosanitary monitoring also showed persistent disease pressure across cacao-producing districts of Amazonas; frosty pod rot remained the most prevalent disease, although its regional mean decreased from 96.3% in 2021 to 65.4% in 2024, while witches’ broom declined from 42.4% to 28.5%. Overall, the integration of environmental suitability, territorial restrictions, restoration opportunities, and phytosanitary conditions provides a spatial basis for prioritizing cacao production in areas that combine productive potential with conservation and deforestation-free requirements.
